# Sickness absenteeism among nurses after the COVID-19 pandemic: A study protocol

**DOI:** 10.1371/journal.pone.0314763

**Published:** 2025-02-13

**Authors:** Halim Ismail, Zhe Shen Huam, Sheng Qian Yew, Hanis Ahmad, Chan Chee Hoong David, Mohd Hafiz Baharudin, Nor Azila Muhd Aris

**Affiliations:** Department of Public Health Medicine, Faculty of Medicine, Universiti Kebangsaan Malaysia, Kuala Lumpur, Malaysia; Faculty of Nursing, Port Said University, EGYPT

## Abstract

**Introduction:**

Sickness absenteeism among the nurses in Malaysia is not fully understood. Complicated with the increase in workload and mental stress during the COVID-19 pandemic and the manifestation of long COVID-19 symptoms, there is a need for an updated insight on the prevalence and the risk factors of sickness absenteeism among nurses in Malaysia. As such, we designed a study protocol that assess the prevalence and risk factors of sickness absenteeism among nurses in Malaysia in the post-COVID-19 pandemic era.

**Materials and methods:**

This is a correlational cross-sectional study. The sociodemographic and clinical questionnaire, sickness absenteeism questionnaire, job characteristics questionnaire, Demand-Control-Support Questionnaire (DCSQ), Work-Related Strain Inventory (WRSI), Work and Family Conflict Scale (WAFCS), the COVID-19-related workplace worries questionnaire, as well as the Depression, Anxiety, and Stress (DASS-21) questionnaire will be randomly distributed to 166 nurses from October 2024 to May 2025.

**Discussion:**

While physical illnesses, psychological disorders, job-related factors, and sociodemographic factors have been identified as risk factors to sickness absenteeism among healthcare professionals in general, the role of these risk factors in causing sickness absenteeism among the nurses remains unclear. Additionally, the increased stress and workload faced by nurses during the COVID-19 pandemic, as well as post-acute COVID-19 syndrome, may have further impacted sickness absenteeism.

**Conclusion:**

By examining the various risk factors of sickness absenteeism, especially in the post-COVID-19 pandemic era, this research will inform future targeted interventions to reduce sickness absenteeism among Malaysian nurses and its associated consequences.

## Introduction

Sickness absenteeism represents a significant occupational health challenge within the healthcare system [1]. It refers to the absence from work resulting from occupational illnesses and/or injuries among the employees [2]. In the healthcare industry, sickness absenteeism imposes a substantial economic burden on healthcare institutions and communities in terms of costs and productivity. A study conducted in Mongolia by Sukhee et al. revealed that absenteeism among health care workers led to an average loss of salary amounting to USD 34.7, an average loss of productivity worth USD 129.8, and an average compensation of USD 130.9, culminating in an average cost of USD 295.5. These figures translated to an estimated total loss of USD 1.8 million across the public health care institutions annually [1]. In terms of productivity, sickness absenteeism may further diminish the healthcare workforce in Malaysia, which has long struggled with staff shortages [3]. This issue is particularly critical among nurses, with a projected shortage nearing 60% by 2030 [4].

Nurses, constituting the largest employee group in the healthcare industry, are undoubtedly most affected by sickness absenteeism. Unfortunately, sickness absenteeism among the nurses is frequently underestimated and understudied [5]. This lack of attention and discussion about sickness absenteeism among nurses could be attributed to the role expectations of the general public towards nurses, who are often expected to be resilient and able to cope with stress and trauma due to the nature of their work [6]. Other factors such as staffing pressures [7] and stigma [8] made the discussion of sickness absenteeism perceived as exacerbating these challenges, leading to underreporting of sickness absenteeism. Previous reports indicated that the prevalence of sickness absenteeism among healthcare workers in general ranges from 26.1% [9] to 41.6% [10]. However, these figures may not be applicable to nurses particularly given that this group of professionals is more heavily engaged in direct patient care during the pandemic (e.g., administering medications, monitoring vital signs, bathing the patients, administering vaccines, etc), which will expose them to additional biological and physical hazards. Of note, a local study reported that prevalence of absenteeism was 78% among the nurses in Malaysia. Nonetheless, this is actually the involuntary job absenteeism and a majority of these absenteeism were in fact due to emergency leaves for multiple reasons but not due to occupational illnesses and/or injuries (i.e. sickness absenteeism) alone [11]. As such, the true extent and severity of sickness absenteeism among the nurses in Malaysia is still unknown.

Although the risk factors of sickness absenteeism among nurses are understudied, prior studies have shown that sickness absenteeism among healthcare professionals is a multifactorial phenomenon influenced by various factors [12–15]. Direct risk factors of sickness absenteeism include physical illnesses (e.g., musculoskeletal diseases, connective tissue disorders, and respiratory diseases) and psychological disorders (e.g., depression, anxiety, and stress) [12]. Additionally, indirect risk factors such as organisational factors (e.g., nature of work, cultural expectations, high work demands, long working hours, night shifts, changing rostering patterns, workload, and poor social support) [13] and sociodemographic factors [14] can influence and exacerbate the direct risk factors of sickness absenteeism. Considering the increased workload, mental stress, exhaustion, and mortality experienced by nurses during the COVID-19 pandemic, complicated with the manifestation of post-acute COVID 19 syndrome after the infection [15], the above risk factors of sickness absenteeism could be relevant and applicable among the nurses. Given the above gaps in literature, this study aims to address the following research questions: (i) What is the prevalence of sickness absenteeism among nurses in Malaysia in the post-COVID-19 pandemic era” is an important research question that need to be addressed. (ii) Is there any association between sociodemographic and clinical factors, job characteristics, job demand, control, and support, work-related strain and stress, work and family conflicts, workplace worries, as well as psychological illnesses with sickness absenteeism among nurses in Malaysia in the post-COVID-19 pandemic era?

To address the aforementioned research questions, this study aims to assess the prevalence and risk factors of sickness absenteeism among nurses in Malaysia in the post-COVID-19 pandemic era. Specifically, we will determine the prevalence of sickness absenteeism among nurses in Malaysia in the post-COVID-19 pandemic era. Then, we will determine the association between (i) sociodemographic and clinical factors, (ii) job characteristics, (iii) job demand, control, and support, (iv) work-related strain and stress, (v) work and family conflicts, (vi) workplace worries, as well as (vii) psychological illnesses with sickness absenteeism among nurses in Malaysia in the post-COVID-19 pandemic era.

## Materials and methods

### Study design

The present study utilises a correlational cross-sectional study design.

### Study setting

This study will be carried out at a teaching hospital located in Kuala Lumpur, Malaysia. This hospital holds a major position as a referral center that deliver advanced tertiary care to patients tested positive for COVID-19 during the pandemic.

### Study population

Nurses employed across various departments in the teaching hospital (i.e., general medicine, general surgery, orthopaedics, obstetrics and gynaecology, paediatrics, emergency department, other specialties) during the pandemic, irrespective of their position or responsibilities, will be eligible to participate in the study. Furthermore, they must have accumulated a minimum of one month of direct occupational exposure to patients diagnosed with or suspected of having COVID-19 infection throughout the pandemic. However, nurses who are on extended leaves such as study leaves, maternity leaves, or special leaves at the time of recruitment will not be included in the study.

### Sample size estimation

The sample size for the study was determined using Kish’s formula sample size calculation for finite population. Given the prevalence of sickness absenteeism among nurses = 41.6% [10], confidence level = 95%, margin of error = 5%, and the total number of nurses in the teaching hospital = 300, the estimated sample size required for the study was 166 participants. The estimated number of nurses in each of the clinical departments and the required samples from each department is tabulated in Table 1 below.

**Table 1:** The estimated number of nurses from each of the department in the teaching hospital and the required samples from each department.

Departments	Estimated Number of Nurses, n (%)	Required Samples from Each Department, n
General Medicine	50 (16.7)	27
General Surgery	50 (16.7)	27
Orthopaedics	30 (10.0)	17
Obstetrics and Gynaecology	30 (10.0)	17
Paediatrics	30 (10.0)	17
Emergency	40 (13.3)	23
Psychiatry	35 (11.7)	19
Anaesthesiology	35 (11.7)	19
Total	300 (100.0)	166

### Sampling technique

The researchers will begin by obtaining a list from the human resources department, known as the sampling frame, which includes all nurses working across different departments. Each nurse in the sampling frame (i.e., sampling unit) will be assigned a unique number from 1 to 300. Using an online random number generator, 166 random numbers will be selected, and the nurses corresponding to these numbers will be chosen. These 166 nurses will then be screened to confirm they meet all eligibility criteria. Nurses who qualify will receive a Google Form via email and/or WhatsApp, which includes the study objectives, instructions, questionnaires, and informed consent form. If any selected nurse does not meet the eligibility requirements, an additional random number will be generated to maintain the target sample of 166. The study will be conducted from October 2024 to May 2025.

### Study instruments

**Fig 1:**
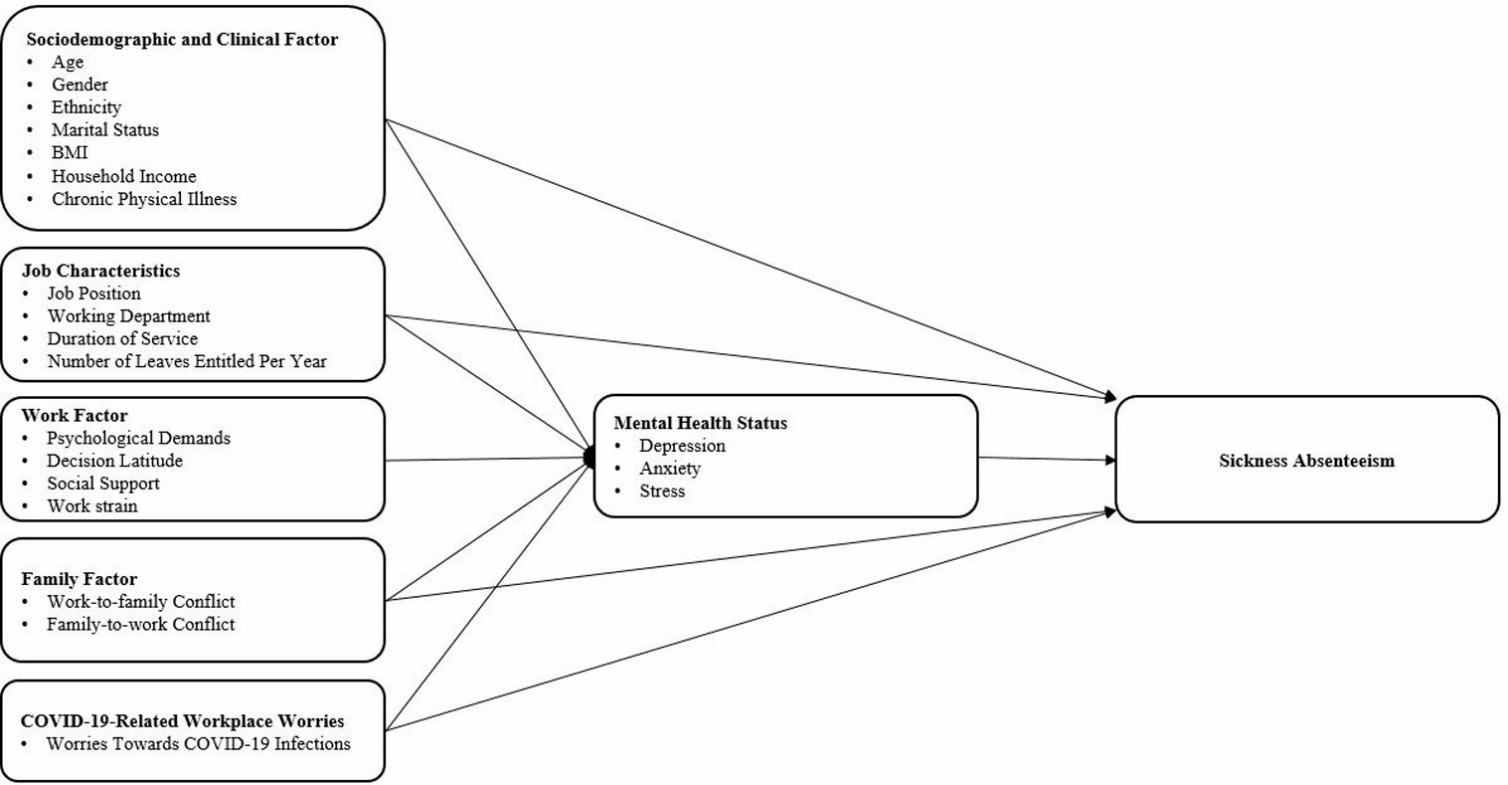
Conceptual framework of the study.

In this study, a self-administered questionnaire (in English language) in the format of Google form will be distributed to the eligible participants. This questionnaire will be developed based on the conceptual framework that is modified from the JOINT Model of Nurse Absenteeism and Turnover [16]. This as illustrated below (Fig 1).

This questionnaire consists of eight sections, including the (i) sociodemographic and clinical data questionnaire, (ii) sickness absenteeism questionnaire, (iii) job characteristics questionnaire, (iv) Demand-Control-Support Questionnaire (DCSQ), (v) Work-Related Strain Inventory (WRSI), (vi) Work and Family Conflict Scale (WAFCS), (vii) the sources of COVID-19-related workplace worries questionnaire, as well as (viii) the Depression, Anxiety and Stress Scale 21-item (DASS-21) questionnaire. Only Section (i) to Section (iii) of the questionnaire are self-developed, while the rest of the five sections will be adapted fully from previous literatures. Since these adapted questionnaires are not modified nor translated, no validity test is required. However, permission to use these questionnaires will be sought from the respective developer upon study commencement.

In Section (i), a sociodemographic and clinical data questionnaire (Table 2) is formed to collect the sociodemographic and clinical data. This include questions about the participants’ age, gender, race, marital status, body mass index (BMI), household income, and chronic physical illnesses.

**Table 2:** Sociodemographic and clinical data questionnaire.

Items	Operational Definitions	Responses
Age	• Participants’ age at the date of recruitment according to identity card.	• Less than 40 years old • 40 years old and above
Gender	• Participants’ gender according to identity card.	• Male • Female
Ethnicity	• Participants’ ethnicity according to identity card.	• Malay • Non-Malay
Marital status	• Participants’ marriage status at the date of recruitment.	• Single • Divorce or married
BMI	• Self-reported weight (in kilograms) divided by the square of the height (in meters).	• Non-overweight (< 25 kg/m^2^) • Overweight (≥ 25 kg/m^2^)
Household income	• Total accumulated incomes received by members of the household of the participants within a month.	• Less than RM4850 • RM4850 and above
Chronic physical illnesses	• Participants experience at least one physical illness that lasted longer than three months in the past one year.	• Yes • No

In Section (ii), a sickness absenteeism questionnaire (Table 3) is developed to gather information on the number of days of sickness absenteeism in the previous year (i.e., 2022). The participants will need to indicate whether they have any sickness absenteeism in the previous year using a “yes” or “no” dichotomous response. If yes, they will need to report the number of days of sickness absenteeism.

**Table 3:** Sickness absenteeism questionnaire.

Items	Operational Definitions	Responses
Experience of sickness absenteeism	• Participants to indicate whether they have taken any sick leave in the past one year	• Yes • No
Number of days of sickness absenteeism	• If answer “yes” to the above item, participants need to report the number of days they have taken sick leave in the past one year	• Number of days of sickness absenteeism

In Section (iii), a job characteristics questionnaire (Table 4) is constructed to collect responses regarding the participants’ job positions, working departments, duration of services, working hours, and the number of leaves entitled in a year. Job positions are categorised as administrative group (i.e., matron and sister) and clinical group (e.g., staff nurse). Meanwhile, working departments are categorised as medical fields (i.e., general medicine, psychiatry, paediatrics, emergency, and anaesthesiology) and surgical fields (e.g., orthopaedics, obstetrics and gynaecology, as well as general surgery). Duration of services is categorised as “less than 10 years” and “10 years and above”. Working hours is categorised as “less than 45 hours” and “45 hours and above”. Number of leaves entitled in a year is categorised into “less than 30 days” and “30 days and above”.

**Table 4:** Job characteristics questionnaire.

Items	Operational Definitions	Responses
Job positions	• Participants’ day-to-day responsibilities	• Administrative group (e.g., matron or sister) • Clinical group (e.g., staff nurse)
Working department	• Department at which the participants are working during the recruitment period	• Medical field (e.g., general medicine, psychiatry, paediatrics, emergency, and anaesthesiology) • Surgical field (e.g., orthopaedics, obstetrics and gynaecology, and general surgery)
Duration of services	• Duration of working experiences (in years) at the hospital	• Less than 10 years • 10 years and above
Working hours	• Duration of working (in hours) per week	• Less than 45 hours • 45 hours and above
Number of leaves entitled per year	• The number of days of paid leave in a year that the participant is entitled to take	• Less than 30 days • 30 days and above

In Section (iv), the Demand-Control-Support Questionnaire (DCSQ) will be used in the present study to measure the job demand, control and support among the nurses. It consists of 17 items distributed across three subscales (i.e., psychological demands, decision latitude, and social support) [17]. Participants are required to answer each item using a four-point Likert scale, ranging from 1 (“often”) to 4 (“never”). The scores of all items in each subscale are added. Cut-off scores of ≥10 points, ≥12 points, and ≥10 points will be set for the psychological demands, decision latitude, and social support subscales, respectively. Participants who score above these cut-off scores indicate that they have high psychological demands, high decision latitude, and high social support at work. The internal consistency of all subscales in the DCSQ was found to be satisfactory, with a Cronbach’s alpha of ≥ 0.72 and item-total correlations ≥ 0.33. Additionally, in term of construct validity, exploratory factor analysis supported the theoretical framework of the DCSQ construct, as the 17 items were loaded onto three factors as expected [18].

In Section (v), the 18-item Work-Related Strain Inventory (WRSI) is adapted to determine the work-related strain and stress among the nurses [19]. Participants need to answer all items using a four-point Likert response, ranging from 1 (“does not apply to me”) to 4 (“does apply to me”). Participants who score ≥36 points are considered having high work strain. The internal consistency of all subscales in the WRSI ranged 0.85–0.90 (i.e., good internal consistency). The test-retest correlation was 0.63 [19]. For concurrent validity, the scores of WRSI were significantly correlated with the subscale scores of the Maslach Burnout Inventory (MBI) (r = 0.2–0.64, p < 0.05).

In Section (vi), the 10-item Work and Family Conflict Scale (WAFCS) will also be adapted into the present study to measure the severity of work and family conflicts among the nurses [20]. It consists of two subscales, which are the “work-to-family conflict” (WFC) and “family-to-work conflict” (FWC) subscales. The participants have to answer each item using a seven-point Likert scale, ranging from 1 (“very strongly disagree”) to 7 (“very strongly agree”). Participants who scored ≥20 points for WFC and/or FWC are considered having high level of family conflict. In terms of reliability, the coefficients H for both subscales of WAFCS were 0.91, indicating very good internal consistency. With regards to concurrent validity, the WFC subscale correlated highly with the Frone’s WFC subscale (r = 0.82) and the FWC subscale correlated highly with the Frone’s FWC subscale (r = 0.73). In term of construct validity, both exploratory factor analysis and confirmatory factor analysis suggest a two-factor structure [20].

In Section (vii), to identify the workplace worries of the nurses during the COVID-19 pandemic, we will adapt the COVID-19-related workplace worries questionnaire [21]. It consists of six items, including the nurses’ worries of getting infected, infecting others, getting quarantined, inadequate personal protective equipment (PPE), inadequate training, and workload. Participants are required to indicate how often they are affected by the different sources of workplace worries using a five-point Likert scale, ranging from 0 (“none”) to 4 (“all of the time”). Participants who score ≥12 points are considered having work-related worries during the COVID-19 pandemic.

In Section (viii), the Depression, Anxiety and Stress Scale 21-item (DASS-21) questionnaire is a 21-item self-administered scale to measure depression, anxiety, and stress concurrently [22]. It has three subscales, namely depression, anxiety, and stress. It is adapted in the present study to identify psychological disorders experienced by the nurses in the past one year. Participants need to rate each item using a 4-point Likert scale from 0 (“did not apply to me at all”) to 3 (“applied to me very much or most of the time”). Cut-off scores of ≥5 points, ≥4 points, and ≥8 points will be set for the depression, anxiety, and stress subscale, respectively. Participants who score above these cut-off scores indicate that they have depression, anxiety, and stress. Cronbach’s alpha value for the overall DASS-21 scale, depression, anxiety, and stress were 0.96, 0.92, 0.87, and 0.89, respectively, which indicate good to excellent internal consistency. The inter-item correlations for each subscale indicated acceptable discrimination (i.e., 0.69–0.80 for depression, 0.44–0.78 for anxiety, and 0.61–0.80 for stress). However, in term of construct validity, the confirmatory factor analysis suggest a one-factor structure [23].

Reminders will be sent to the participants if they do not respond to the questionnaires within a month. To avoid missing data, the Google form will be pre-set in such a way that answers to all questions is mandatory prior to submission. To avoid duplicate responses, the researchers will activate the “limit to one response” setting so that each participant can only participate once.

### Statistical analyses

The data will be analysed using the Statistical Package for Social Sciences (SPSS) Statistics version 26. Categorical data such as the sociodemographic and clinical data as well as the job characteristics will be analysed using frequencies and percentages. Meanwhile, continuous data including the number of sickness absenteeism, work demand, control, and support, work-related strain and stress, work and family conflicts, as well as the workplace worries will be analysed using means and standard deviations.

The prevalence of sickness absenteeism will be presented in percentage. Meanwhile, the association between independent variables (i.e., sociodemographic and clinical data, job characteristics, work strain and stress, work-related worries with sickness absenteeism, job demand, control, and support, work and family conflicts, as well as psychological illnesses) with dependent variable (i.e., sickness absenteeism) will be analysed using simple logistic regression. Independent variables with p<0.25 will be modelled into a multiple logistic regression using forward method to determine the significant risk factors of sickness absenteeism.

### Ethical considerations

The present study has been approved by the Ethics Committee of the National University of Malaysia (JEP-2023-239). Information regarding the objectives of the study will be provided to the potential participants. Informed consent from the participants shall be obtained digitally. To ensure anonymity during data collection, each nurse will be assigned a unique identification code instead of personal identifiers such as names or employee numbers. During data analysis, all electronic data will be securely stored on password-protected, encrypted drives or servers, accessible only to authorized researchers with encryption keys. Furthermore, data will be analysed and presented in aggregated form rather than focusing on individual cases or specific departments. This approach minimises the risk of identifying individuals or departments with particularly high or low rates of sickness absenteeism. All procedures in data collection and analyses will adhere to the Declaration of Helsinki.

## Discussion

In 2018, Malaysia had over a hundred thousand nurses serving both the public and private sectors [24]. These dedicated health care professionals not only provide medical services to patients but also engage in health promotion activities such as providing patient education, health screenings, and other health-related tasks to the community. Since nurses are involved with a variety of daily tasks, it comes as no surprise that are more exposed to physical and mechanical hazards due to frequent bending, stretching, lifting, and moving of patients [25]. Moreover, nurses constantly come into contact with potentially biological hazards, including medications, radiation, infectious diseases, needlesticks, and cleaning chemicals [25]. Consequently, nurses experience a high rate of sickness absenteeism within the health care workforce [11], leading to substantial monetary and societal costs [1]. Given the significant impact of sickness absenteeism on nurses and the associated costs, it is essential to understand the prevalence and risk factors of sickness absenteeism in this population.

Physical illnesses and psychological disorders are known contributors of sickness absenteeism among the nurses, potentially influenced by job-related and sociodemographic factors [12–14]. However, the role and relationship of other risk factors of sickness absenteeism in the general population, such as family factors [26] and psychological well-being [27], remains unclear in the context of sickness absenteeism among the nurses. Additionally, with the majority of nurses being mobilised to provide care for COVID-19 patients during the pandemic, it is crucial to investigate how the increased stress and workload, as well as the long COVID-19 symptoms [15], affect sickness absenteeism among nurses in the post-pandemic era. As such, this study will address these research gaps and shed light on the risk factors of sickness absenteeism in Malaysian nurses.

One of the major challenges in this study is the potential for recall bias. To mitigate this bias, participants will be encouraged to refer to their leave records available on the hospital’s staff portal, rather than relying solely on memory. Moreover, there is a risk of response bias where participants may provide answers that they believe will be favourable to the researcher, such as underreporting of sickness absenteeism, underreporting of stress levels, or reluctance to disclose personal or family issues. To minimise this bias, data confidentiality will be ensured and participants will be assured that their responses will remain anonymous. Since the questionnaires will be distributed digitally through Google forms (i.e., without face-to-face contact), there is a possibility of non-response bias, as some participants may refuse to participate or show disinterest in the data collection exercise. To address this, a long data collection period lasting three months will be implemented, and regular reminders will be sent to participants via email and/or Whatsapp contacts.

Besides the risk factors presented in the methods, there might be other confounders that might affect the association between the risk factors and sickness absenteeism among nurses. Efforts will be taken to control for these confounders using multiple logistic regression upon data analysis. Furthermore, although the cross-sectional study design is unable to establish causal-relationship of sickness absenteeism, it enables the identification of the prevalence and risk factors of sickness absenteeism rapidly, given the limitations in time and budget. Finally, we acknowledge that there are limitations to conducting research at a single site (i.e., a teaching hospital in Kuala Lumpur that served as a referral center for COVID-19 patients during the pandemic), such that the generalisability of findings may be limited to the entire nurse population in Malaysia. Nonetheless, the finding from the present study will inform our multicenter research in future.

## Conclusion

In conclusion, the present study aims to provide insight on the prevalence and risk factors of sickness absenteeism among nurses in Malaysia. By examining the various risk factors of sickness absenteeism, especially in the post-COVID-19 pandemic era, this research will inform future interventions to reduce sickness absenteeism among Malaysian nurses and its associated consequences.
